# Extracellular vesicle Cystatin C and CD14 are associated with both renal dysfunction and heart failure

**DOI:** 10.1002/ehf2.12699

**Published:** 2020-07-10

**Authors:** Laura Verbree‐Willemsen, Ya‐nan Zhang, Irwani Ibrahim, Shirley B.S. Ooi, Jiong‐Wei Wang, Muhammad I. Mazlan, Win S. Kuan, Siew‐Pang Chan, Linda M. Peelen, Diederick E. Grobbee, A. Mark Richards, Carolyn S.P. Lam, Dominique P.V. de Kleijn

**Affiliations:** ^1^ Julius Center for Health Sciences and Primary Care University Medical Center Utrecht, Utrecht University Utrecht The Netherlands; ^2^ Department of Surgery, Yong Loo Lin School of Medicine National University of Singapore Singapore; ^3^ Cardiovascular Research Institute National University Heart Centre Singapore Singapore; ^4^ Department of Emergency Medicine National University Health System Singapore Singapore; ^5^ Department of Medicine, Yong Loo Lin School of Medicine National University of Singapore Singapore; ^6^ Department of Anaesthesiology University Medical Center Utrecht, Utrecht University Utrecht The Netherlands; ^7^ Christchurch Heart Institute University of Otago Christchurch New Zealand; ^8^ National Heart Centre Singapore, Duke‐NUS Graduate Medical School Singapore; ^9^ Department of Cardiology University Medical Center Groningen Groningen The Netherlands; ^10^ Department of Vascular Surgery University Medical Center Utrecht, Utrecht University Utrecht The Netherlands; ^11^ Netherlands Heart Institute Utrecht University Utrecht The Netherlands

**Keywords:** Heart failure, Renal insufficiency, Extracellular vesicles, Cardiorenal syndrome, CD14, Cystatin C

## Abstract

**Aims:**

Extracellular vesicles (EVs) are small double‐membrane plasma vesicles that play key roles in cellular crosstalk and mechanisms such as inflammation. The role of EVs in combined organ failure such as cardiorenal syndrome has not been investigated. The aim of this study is to identify EV proteins that are associated with renal dysfunction, heart failure, and their combination in dyspnoeic patients.

**Methods and results:**

Blood samples were prospectively collected in 404 patients presenting with breathlessness at the emergency department at National University Hospital, Singapore. Renal dysfunction was defined as estimated glomerular filtration rate < 60 mL/min/1.73 m^2^. The presence of heart failure was independently adjudicated by two clinicians on the basis of the criteria of the European Society of Cardiology guidelines. Protein levels of SerpinG1, SerpinF2, Cystatin C, and CD14 were measured with a quantitative immune assay within three EV sub‐fractions and in plasma and were tested for their associations with renal dysfunction, heart failure, and the concurrence of both conditions using multinomial regression analysis, thereby correcting for confounders such as age, gender, ethnicity, and co‐morbidities. Renal dysfunction was found in 92 patients (23%), while heart failure was present in 141 (35%). In total, 58 patients (14%) were diagnosed with both renal dysfunction and heart failure. Regression analysis showed that Cystatin C was associated with renal dysfunction, heart failure, and their combination in all three EV sub‐fractions and in plasma. CD14 was associated with both renal dysfunction and the combined renal dysfunction and heart failure in all EV sub‐fractions, and with presence of heart failure in the high density lipoprotein sub‐fraction. SerpinG1 and SerpinF2 were associated with heart failure in, respectively, two and one out of three EV sub‐fractions and in plasma, but not with renal dysfunction.

**Conclusions:**

We provide the first data showing that Cystatin C and CD14 in circulating EVs are associated with both renal dysfunction and heart failure in patients presenting with acute dyspnoea. This suggests that EV proteins may be involved in the combined organ failure of the cardiorenal syndrome and may represent possible targets for prevention or treatment.

## Introduction

Heart failure and renal failure commonly coexist: patients with heart failure have a higher chance of developing renal failure and vice versa.^1^, ^2^ Declines in renal function are associated with development of ventricular dysfunction and worsening prognosis in heart failure.^3^, ^4^ Such adverse interactions between the heart and kidneys comprise the cardiorenal syndrome (CRS).^1^, ^2^ However, the underlying pathophysiological mechanisms in cardiorenal crosstalk are not fully understood.^1^ Identifying common underlying mechanisms affecting both organs will help elucidate the pathophysiology of CRS.

Plasma extracellular vesicles (EVs) are small double‐membrane vesicles abundant in plasma.^5^ EVs are important in communication between cells and are involved in key processes including inflammation and coagulation.^5^, ^6^ EVs are produced by all cell types and contain proteins, (micro)RNA, and surface molecules from their parent cells.^5^ The release of EVs increases under hypercoagulable and inflammatory conditions, and the number and content of EVs have been associated with cardiovascular diseases.^6^, ^7^, ^8^ Pertinent to cardiorenal crosstalk, it has been reported that EVs released from one organ may induce dysfunction in remote organs.^9^

Previously, we reported that EV protein levels of Cystatin C, CD14, SerpinG1, and SerpinF2 were associated with heart failure in dyspnoeic patients.^10^ Given the relevance of renal dysfunction, inflammation, and the coagulation system in the development of cardiovascular diseases, we extended previous work on Cystatin C, CD14, SerpinG1, and SerpinF2 within circulating EVs to explore their possible associations with the CRS. Cystatin C is a marker for renal dysfunction, and it has been associated with increased risk of cardiovascular events.^11^, ^12^ CD14 is a pro‐inflammatory protein involved in the innate immune system and serves as co‐receptor for toll‐like receptors.^13^ SerpinG1 is also known as C1‐inhibitor and inhibits several proteases in the complement, coagulation, and fibrinolysis systems.^14^ SerpinF2, also known as α2‐antiplasmin, is the main inhibitor of plasmin and thereby reduces fibrinolysis.^15^ EV levels of these four proteins have been associated with heart failure, but their role in cardiorenal crosstalk has not been investigated. EV proteins associated with both conditions may indicate that these proteins are involved in aetiology of both diseases and point to associated signalling pathways as possible targets for future prevention and treatment options.

The primary aim of this study is to investigate the association between the selected EV proteins (Cystatin C, CD14, SerpinG1, and SerpinF2) with both renal dysfunction and heart failure in patients with acute dyspnoea.

## Methods

This is an observational study in 404 patients presenting with recent onset breathlessness at the Emergency Department of the National University Hospital Singapore. This is a sub‐analysis of the Biomarkers Beyond BNP in Breathlessness (“4B”) study, a prospective, single‐centre cohort study. The study was approved by the Domain Specific Review Board of National Health Group of Singapore (reference number: 2012/0090) and conforms to the Declaration of Helsinki. Written informed consent was obtained from all patients.

### Study population

Patients attending the Emergency Department of the National University Hospital Singapore during daytime between 2010 and 2013 with the primary complaint of shortness of breath were included in the 4B study. Patients who required immediate intensive care or intubation and patients who were unable or unwilling to give informed consent were not included. Exclusion criteria were age under 21 years, shortness of breath related to trauma, and current haemodialysis. In total, 607 patients were recruited, and owing to sample availability, 404 are included in the current analysis.

### Data collection

On admission to the emergency department, blood was drawn and information on patient history, symptoms, and clinical findings was collected according to a standardized protocol. All blood samples were collected in EDTA tubes and spun at 4000 *g* for 10 min at 4°C to obtain plasma. Plasma samples were stored at −80°C prior to further analysis. EVs were precipitated in sub‐fractions from the plasma according to a uniform protocol. After precipitation, the EVs were lysed in order to release their content, and four empirically selected proteins were measured quantitatively with commercial immunoassays. Those undertaking the laboratory procedures were blinded to the adjudicated diagnoses.

### Isolation of extracellular vesicle plasma sub‐fractions

Precipitation of low‐density lipoprotein (LDL) and high‐density lipoprotein (HDL) plasma fractions was performed as previously described.^10^ In the supplementary data of Zhang *et al*.^10^ they showed by using electron microscopy, density gradient centrifugation, and subsequent CD9 Western blotting and immunobead assay that CD14, SerpinF2, SerpinG1, and Cystatin C are in the EV fraction. For SerpinF2, this is also shown in our manuscript in the *International Journal of Molecular Sciences*.^16^ For detailed methods of precipitation, we refer to Zhang and Wang; in brief, EVs were isolated from three precipitates: (i) the total EV (TEX) fraction was isolated from the precipitate of 125 μL of EDTA plasma. The precipitated pellet was dissolved in 125 μL of Roche lysis buffer and used in the quantitative magnetic bead assays as the TEX fraction. (ii) In another 125 μL EDTA plasma, LDL was precipitated using MnCl_2_ and dextran sulfate. The LDL pellet with the co‐precipitated EVs was dissolved in 125 μL of Roche lysis buffer and used in the quantitative magnetic bead assays as the LDL fraction. (iii) The supernatant was used to precipitate the HDL fraction and the co‐precipitated EVs by increasing the MnCl_2_ and dextran sulfate concentration in the LDL supernatant. The pellet was dissolved in 125 μL of Roche lysis buffer and used in the quantitative magnetic bead assays as the HDL fraction.

### Quantitative protein assay

Quantitative analysis of the selected proteins was performed as previously described.^10^ In brief, MagPlex‐C Microspheres (Luminex #MC100xx‐01) were conjugated with the selected antibodies to provide bead‐capture antibody complexes. Samples were incubated with the bead‐capture antibody complex and subsequently with the biotinylated antibodies to detect the captured protein. Streptavidin–phycoerythrin (SA‐PE; BD Biosciences #554061) was added to quantify the concentration of captured protein. Standard curves were correlated with PE signal and dilution of homologous recombinant proteins. The Bio‐Plex1 200 Systems (Bio‐Rad #171–000201) was used for readout and data processing. Individual concentrations of CD14, SerpinG1, Cystatin C, and SerpinF2 proteins in all three EV sub‐fractions (HDL, LDL, and TEX sub‐fraction) and in plasma were used in the current analyses.

### Outcomes

Endpoints for this study were the presence of heart failure and renal dysfunction (categorical variables) and estimated glomerular filtration rate (eGFR) and N‐terminal pro‐brain natriuretic peptide (NT‐proBNP) concentrations (continuous variables). The diagnosis of heart failure was adjudicated by two clinicians [an emergency department specialist (S. O.) and cardiologist (A. M. R.)] according to the European Society of Cardiology criteria.^17^ Adjudicators independently reviewed all available information on each patient and attributed breathlessness to heart failure or another cause. They could access medical records, case report forms, and other test results including cardiac imaging, if available, but were blinded to plasma concentrations of NT‐proBNP and multiple other markers assayed as part of the main 4B study objectives.^18^ In case of disagreement between the two adjudicators, a third adjudicator gave the final diagnosis. Renal dysfunction was defined as eGFR < 60 mL/min/1.73 m^2^.

### Potential confounding factors

Age, gender, ethnicity, diabetes, hypertension, and ischaemic heart disease (IHD) were considered as potential confounders and therefore included in multivariable analyses. IHD was defined as history of coronary artery disease, myocardial infarction, percutaneous coronary intervention, or coronary artery bypass grafting as indicated by the patient and verified in medical records.

### Data analysis

Baseline characteristics were analysed for the entire cohort and for four separate patient groups: (i) patients with no renal dysfunction and no heart failure, (ii) patients with heart failure without renal failure, (iii) patients with renal dysfunction without heart failure, and (iv) patients with both conditions concurrently. Descriptive statistics employed included mean values with standard deviations and medians with interquartile ranges (25th to 75th percentiles) calculated for continuous variables, normally and non‐normally distributed, respectively. Categorical data were displayed in percentages.

Median levels of Cystatin C, CD14, SerpinG1, and SerpinF2 in the (HDL, LDL, and TEX) EV sub‐fractions and in plasma were compared between patients with heart failure and/or renal dysfunction using Kruskal–Wallis tests. To present median protein levels graphically, standardized median protein levels were calculated by dividing the medians of patients with heart failure, renal dysfunction, and the combination of both conditions by the median of patients with no heart failure and no renal dysfunction.

The sample concentrations of Cystatin C, CD14, SerpinG1, and SerpinF2 in all EV sub‐fractions and in plasma were tested for associations with heart failure and renal dysfunction using univariable and multivariable logistic regression analyses. We also examined the association between the protein concentrations and continuous levels of NT‐proBNP and eGFR using linear regression analyses. After separate consideration of renal dysfunction and heart failure, we repeated the analyses for the combination of these two diagnoses, using a categorical outcome variable including the same four categories as described above: (i) no renal dysfunction and no heart failure, (ii) heart failure without renal failure, (iii) renal dysfunction without heart failure, or (iv) the combination of both conditions. The association between the protein levels and this categorical endpoint was analysed using multinomial regression analyses.

In all univariable and multivariable regression analyses, protein levels were transformed using log transformation for Cystatin C, CD14, SerpinG1, and NT‐proBNP and square root transformation for SerpinF2 in order to fulfil the assumptions of normality and homoscedasticity. Transformed protein levels were then standardized by dividing the centred protein levels by their standard deviations to compare the effect sizes of different proteins with each other. The following variables were considered as potential confounders and therefore included in the multivariable analyses: age, gender, ethnicity, diabetes, hypertension, and IHD.

For the analyses addressing renal dysfunction and heart failure as separate outcomes, we corrected for multiple testing using the Bonferroni method resulting in a level of significance of 0.025. For the multinomial analyses, level of significance of 0.05 was used. Results are presented as beta coefficients for the linear regression analyses and odds ratios (ORs) for the logistic regression models, with their accompanying 97.5% or 95% confidence intervals. All statistical analyses were performed in Rstudio using R software for statistical computing version 3.4.2.^19^

## Results

The study sub‐cohort analysed comprised 404 patients with a mean age of 56 years and differing ethnic background. Half were Chinese, 28% Malay, and 17% Indian. Differences in baseline characteristics in patients with heart failure and/or renal dysfunction are shown in *Table* *1*.

**Table 1:** Baseline characteristics

	All (*n* = 404)	No HF/RD (*n* = 222)	Only HF (*n* = 83)	Only RD (*n* = 34)	HF + RD (*n* = 58)
Age in years, mean (SD)	55.9 (14.5)	51.4 (14.4)	57.5 (9.7)	66.3 (12.2)	67.0 (10.8)
Male gender	268 (66)	142 (64)	67 (81)	19 (56)	38 (66)
Patient ethnicity					
Chinese	195 (48)	104 (47)	39 (47)	17 (50)	32 (55)
Indian	67 (17)	43 (19)	14 (17)	3 (9)	7 (12)
Malay	112 (28)	58 (26)	25 (30)	12 (35)	14 (24)
Other	30 (7)	17 (8)	5 (6)	2 (6)	5 (9)
Body mass index, median (IQR)	27 (23–31)	26 (23–31)	27 (24–33)	28 (25–30)	27 (23–30)
Current or past smoker	118 (29)	65 (30)	34 (41)	7 (21)	11 (19)
Patient history					
Myocardial infarction	70 (17)	23 (10)	23 (28)	5 (15)	19 (33)
Diabetes	138 (34)	45 (20)	33 (40)	17 (50)	43 (74)
Hypertension	225 (56)	99 (45)	55 (66)	29 (85)	42 (72)
Chronic renal impairment^a^	39 (10)	0 (0)	4 (5)	14 (41)	21 (37)
COPD	25 (6)	14 (6)	5 (6)	2 (6)	4 (7)
Cerebrovascular accident	24 (6)	6 (3)	7 (8)	5 (15)	6 (10)
Congestive heart failure	57 (14)	0 (0)	28 (34)	0 (0)	29 (50)
Ischaemic heart disease^b^	153 (38)	54 (24)	43 (52)	16 (47)	40 (70)
Medication					
Aspirin	116 (29)	41 (18)	37 (45)	4 (12)	34 (60)
Beta‐blocker	121 (30)	41 (18)	35 (42)	7 (23)	38 (69)
Statin	144 (36)	55 (25)	40 (48)	12 (36)	37 (67)
ACE‐inhibitor	71 (18)	16 (7)	29 (35)	7 (23)	19 (35)
Diuretics	78 (19)	7 (3)	33 (40)	5 (15)	33 (60)
Haemoglobin in g/dL, mean (SD)	13.3 (2.0)	13.8 (1.8)	13.6 (2.1)	12.4 (2.0)	11.9 (1.6)

Baseline characteristics are shown for all patients, patients with no heart failure and no renal dysfunction, patients with only heart failure, patients with only renal dysfunction, and patients with both heart failure and renal dysfunction, separately. Values are numbers of patients (with corresponding percentages in parentheses) unless otherwise stated.

Abbreviations: COPD, chronic obstructive pulmonary disease; HF, heart failure; IQR, interquartile range; RD, renal dysfunction; SD, standard deviation.

a Chronic renal impairment was defined as a glomerular filtration rate < 60 mL/min/1.73 m^2^.

b Ischaemic heart disease was defined as a history of coronary artery disease, myocardial infarction, percutaneous coronary intervention, or coronary artery bypass grafting.

### Median protein levels

Median protein levels of Cystatin C, CD14, SerpinG1, and SerpinF2 in patients with heart failure and/or renal dysfunction are shown in *Table* *2*. Median levels of Cystatin C and CD14 were the highest in patients with both heart failure and renal dysfunction in all three EV sub‐fractions. The same was true for plasma Cystatin C, but not for plasma CD14. Differences in median SerpinG1 and SerpinF2 levels were observed in some but not all EV sub‐fractions (*Table* *2*).

**Table 2:** Median protein levels in patients with heart failure and/or renal dysfunction

	No HF/RD (*n* = 222)	Only HF (*n* = 83)	Only RD (*n* = 34)	HF + RD (*n* = 58)	*P*‐value^a^
Cystatin C					
HDL	**35 (26–46)**	**50 (35–67)**	**76 (54–113)**	**89 (61–113)**	**<0.001**
LDL	**152 (89–256)**	**207 (153–325)**	**294 (208–439)**	**348 (213–492)**	**<0.001**
TEX	**535 (376–693)**	**664 (463–937)**	**1243 (801–1721)**	**1446 (1107–2022)**	**<0.001**
Plasma	**2535 (2085–3175)**	**3125 (2413–4212)**	**4410 (2860–7062)**	**4931 (3440–7558)**	**<0.001**
CD14					
HDL	**11 (8–14)**	**13 (10–16)**	**15 (10–18)**	**17 (13–20)**	**<0.001**
LDL	**87 (67–103)**	**86 (71–116)**	**95 (85–139)**	**101 (78–133)**	**0.001**
TEX	**158 (124–204)**	**169 (137–224)**	**197 (166–260)**	**213 (170–289)**	**<0.001**
Plasma	1187 (978–1485)	1185 (971–1331)	1158 (952–1328)	1119 (994–1284)	0.626
SerpinG1					
HDL	193 (140–275)	163 (120–230)	199 (142–259)	188 (133–269)	0.291
LDL	**1156 (781–1767)**	**828 (604–1308)**	**937 (646–1285)**	**679 (448–1076)**	**<0.001**
TEX	**328 (233–511)**	**364 (241–613)**	**297 (197–626)**	**434 (274–686)**	**0.047**
Plasma	**990 (788–1245)**	**1299 (922–2660)**	**921 (748–1318)**	**1216 (917–2133)**	**<0.001**
SerpinF2					
HDL	72 (35–124)	74 (34–131)	89 (39–121)	66 (41–118)	0.932
LDL	**1021 (618–1491)**	**877 (399–1164)**	**935 (619–1579)**	**781 (361–1187)**	**0.012**
TEX	904 (528–1336)	792 (406–1249)	964 (518–1649)	907 (529–1246)	0.237
Plasma	**6247 (3089–9627)**	**4104 (2615–6368)**	**4961 (2548–6815)**	**4901 (2735–7198)**	**0.015**

Abbreviations: HDL, high‐density lipoprotein; HF, heart failure; LDL, low‐density lipoprotein; RD, renal dysfunction; TEX, total extracellular vesicles.

Median protein levels of Cystatin C, CD14, SerpinG1, and SerpinF2 levels in HDL, LDL, and TEX sub‐fractions and in plasma are shown in ng/mL for patients with no heart failure and no renal dysfunction, patients with only heart failure, patients with only renal dysfunction, and patients with both conditions. Values in parentheses are interquartile ranges (25th–75th percentiles).

a *P*‐values result from the comparison median protein levels in the four patients groups, derived from Kruskal–Wallis analysis. Values in bold are statistically significant (*P* < 0.05).

### Heart failure

In total, 141 patients (35%) were diagnosed with heart failure. Cystatin C was significantly associated with increased risk of heart failure in all three EV sub‐fractions and in plasma (*Table* *3*). Higher CD14 levels were also associated with increased risk of heart failure in the HDL and in TEX sub‐fraction. Higher SerpinG1 was directly associated with increased risk of heart failure in TEX sub‐fraction and in plasma, but, inversely associated in the LDL fraction. Higher SerpinF2 levels were also associated with decreased risk of heart failure in LDL sub‐fraction and in plasma (*Table* *3*).

**Table 3:** Association between extracellular vesicle and plasma protein concentrations and heart failure

	Univariable				Multivariable^a^			
	OR	97.5% CI		*P*‐value	OR	97.5% CI		*P*‐value
Cystatin C								
HDL	**2.43**	**1.85**	**3.27**	**<0.001**	**1.86**	**1.38**	**2.56**	**<0.001**
LDL	**2.02**	**1.55**	**2.68**	**<0.001**	**1.63**	**1.22**	**2.23**	**<0.001**
TEX	**2.09**	**1.61**	**2.75**	**<0.001**	**1.61**	**1.19**	**2.20**	**<0.001**
Plasma	**1.97**	**1.47**	**2.72**	**<0.001**	**1.46**	**1.10**	**2.00**	**0.004**
**CD14**								
HDL	**1.94**	**1.49**	**2.56**	**<0.001**	**1.65**	**1.23**	**2.25**	**<0.001**
LDL	**1.28**	**1.01**	**1.63**	**0.021**	1.20	0.91	1.59	0.137
TEX	**1.48**	**1.17**	**1.90**	**<0.001**	**1.38**	**1.04**	**1.83**	**0.012**
Plasma	0.84	0.65	1.07	0.102	0.79	0.59	1.05	0.072
**SerpinG1**								
HDL	0.88	0.70	1.12	0.232	0.89	0.68	1.17	0.335
LDL	**0.57**	**0.43**	**0.74**	**<0.001**	**0.64**	**0.47**	**0.85**	**<0.001**
TEX	**1.34**	**1.06**	**1.71**	**0.006**	**1.50**	**1.14**	**1.99**	**<0.001**
Plasma	**2.03**	**1.52**	**2.76**	**<0.001**	**2.03**	**1.48**	**2.87**	**<0.001**
**SerpinF2**								
HDL	0.97	0.77	1.23	0.788	0.96	0.74	1.25	0.721
LDL	**0.71**	**0.56**	**0.90**	**0.001**	**0.74**	**0.56**	**0.97**	**0.012**
TEX	0.85	0.67	1.07	0.11	0.85	0.64	1.11	0.165
Plasma	**0.70**	**0.54**	**0.91**	**0.003**	**0.66**	**0.48**	**0.88**	**0.001**

Abbreviations: 97.5% CI, 97.5% confidence interval; HDL, high‐density lipoprotein; EV, extracellular vesicle; LDL, low‐density lipoprotein; OR, odds ratio; TEX, total extracellular vesicles.

Results of univariable and multivariable logistic regression analyses for the association between Cystatin C, CD14, SerpinG1, and SerpinF2 levels (in HDL, LDL, and TEX sub‐fractions and in plasma) and heart failure. All protein levels were standardized prior to analyses; shown odds ratios therefore represent odds ratios for one standard deviation increase in the corresponding protein level. Values in bold are statistically significant (*P* < 0.025).

a Corrected for age, gender, ethnicity, diabetes, hypertension, and ischaemic heart disease.

In addition to the binomial outcome of heart failure, we investigated the association between the protein levels and continuous NT‐proBNP levels. These analyses showed similar results. The only difference was that CD14 was now significantly associated with increase in NT‐proBNP levels in all three EV sub‐fractions (*Table* *S1*).

### Renal dysfunction

In total, 92 patients (23%) were classified as having renal dysfunction. As shown in *Table* *4*, both Cystatin C and CD14 were associated with increased risk of renal dysfunction in all three EV sub‐fractions. The ORs were higher for Cystatin C (5.48, 3.35, and 6.18) compared with the ORs for CD14 (1.95, 1.60, and 1.77 in HDL, LDL, and TEX sub‐fractions, respectively). Cystatin C in plasma was also associated with increased risk of renal dysfunction (OR of 2.89), whereas CD14 in plasma was not. Except for SerpinG1 in LDL sub‐fraction, which was associated with decreased risk of renal dysfunction, no other significant associations were found. The same proteins were associated with continuous GFR levels, as shown in *Table* *S2*.

**Table 4:** Association between extracellular vesicle and plasma protein concentrations and renal dysfunction

	Univariable				Multivariable^a^			
	OR	97.5% CI		*P*‐value	OR	97.5% CI		*P*‐value
Cystatin C								
HDL	**6.70**	**4.31**	**11.1**	**<0.001**	**5.48**	**3.32**	**9.81**	**<0.001**
LDL	**3.21**	**2.26**	**4.75**	**<0.001**	**3.35**	**2.17**	**5.47**	**<0.001**
TEX	**9.22**	**5.61**	**16.4**	**<0.001**	**6.18**	**3.69**	**11.3**	**<0.001**
Plasma	**4.05**	**2.71**	**6.37**	**<0.001**	**2.89**	**1.90**	**4.64**	**<0.001**
CD14								
HDL	**2.37**	**1.73**	**3.32**	**<0.001**	**1.95**	**1.34**	**2.93**	**<0.001**
LDL	**1.67**	**1.26**	**2.24**	**<0.001**	**1.60**	**1.13**	**2.29**	**0.003**
TEX	**2.15**	**1.62**	**2.91**	**<0.001**	**1.77**	**1.23**	**2.57**	**<0.001**
Plasma	0.87	0.66	1.14	0.235	0.93	0.66	1.29	0.635
SerpinG1								
HDL	1.07	0.82	1.41	0.565	1.06	0.76	1.47	0.703
LDL	**0.54**	**0.39**	**0.72**	**<0.001**	**0.65**	**0.45**	**0.93**	**0.009**
TEX	1.26	0.96	1.64	0.054	1.15	0.83	1.61	0.335
Plasma	1.18	0.89	1.56	0.183	1.14	0.82	1.59	0.378
SerpinF2								
HDL	0.99	0.76	1.30	0.962	1.02	0.74	1.42	0.880
LDL	0.90	0.69	1.17	0.357	1.00	0.71	1.41	0.974
TEX	1.12	0.86	1.49	0.337	1.16	0.82	1.65	0.343
Plasma	0.81	0.61	1.08	0.108	0.82	0.56	1.17	0.214

Abbreviations: 97.5% CI, 95% confidence interval; EV, extracellular vesicle; HDL, high‐density lipoprotein; LDL, low‐density lipoprotein; OR, odds ratio; TEX, total extracellular vesicles.

Results of univariable and multivariable logistic regression analyses for the association between Cystatin C, CD14, SerpinG1, and SerpinF2 levels (in HDL, LDL, and TEX sub‐fractions and in plasma) and renal dysfunction. All protein levels were standardized prior to analyses; shown odds ratios therefore represent odds ratios for one standard deviation increase in the corresponding protein level. Values in bold are statistically significant (*P* < 0.025).

a Corrected for age, gender, ethnicity, diabetes, hypertension, and ischaemic heart disease.

### Heart failure and renal dysfunction

**Figure 1:**
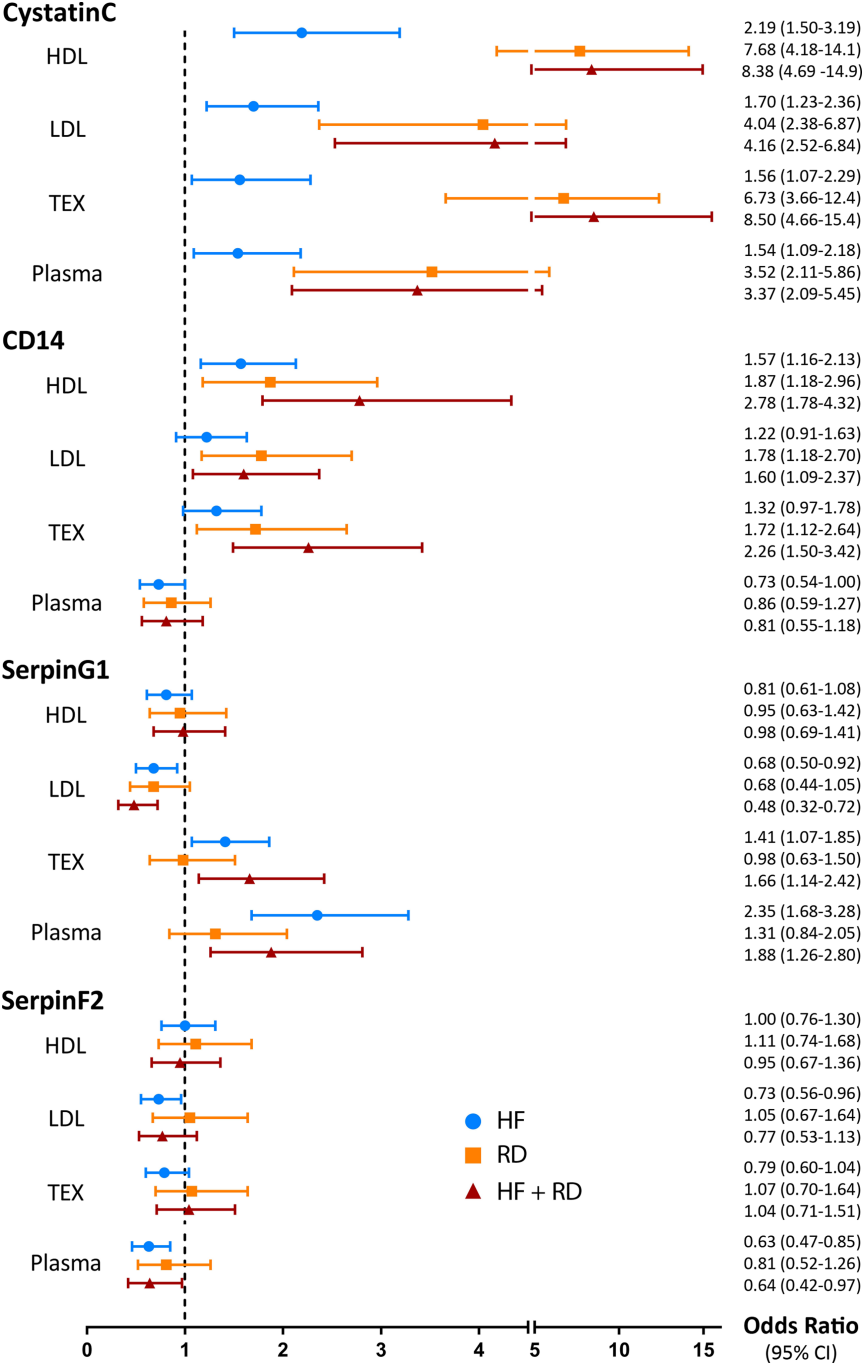
Odds ratios from multinomial regression analyses. Results from multinomial regression analyses for the association between protein levels and heart failure, renal dysfunction, and the combination of both conditions. Odds ratios with corresponding 95% confidence intervals were depicted both as symbols and in numbers for Cystatin C, CD14, SerpinG1, and SerpinF2 in HDL, LDL, and TEX sub‐fractions and in plasma. Patients who did not have heart failure or renal dysfunction were chosen as reference category. All analyses were corrected for age, gender, ethnicity, diabetes, hypertension, and ischaemic heart disease. Abbreviations: 95% CI, 95% confidence interval; HDL, high‐density lipoprotein; HF + RD, heart failure and renal dysfunction; HF, heart failure; LDL, low‐density lipoprotein; RD, renal dysfunction; TEX, total extracellular vesicles.

As shown in *Figure* *1*, Cystatin C was associated with heart failure, renal dysfunction, and the combination of both conditions in all three EV sub‐fractions and in plasma, with the highest ORs for patients with both heart failure and renal dysfunction. Similar results were found for CD14 in EV sub‐fractions. In the HDL fraction, CD14 was associated with an OR of 1.57 [95% confidence interval (95% CI): 1.16–2.13] for heart failure, OR of 1.87 (95% CI: 1.18–2.96) for renal dysfunction, and OR of 2.78 (95% CI: 1.78–4.32) for the combination of both conditions. CD14 was also associated with increased risk of renal dysfunction and the combination of renal dysfunction and heart failure in LDL and TEX sub‐fraction (*Figure* *1*). These results were not found for CD14 in plasma.

SerpinG1 was associated with heart failure, depicted by significant ORs for heart failure and the combination of heart failure and renal dysfunction in LDL and TEX sub‐fraction and in plasma. The same was true for SerpinF2 in plasma. Both SerpinG1 and SerpinF2 were not associated with renal dysfunction in any sub‐fraction or in plasma (*Figure* *1*). Full results of univariable and multivariable multinomial analyses are provided in *Table* *S3*.

## Discussion

In this cross‐sectional cohort study, we showed that EV levels of CD14 and Cystatin C are associated with both renal dysfunction and heart failure in patients presenting with dyspnoea. To our knowledge, this is the first study that has identified EV proteins associated with both heart failure and renal dysfunction, indicating that Cystatin C and CD14 in EVs are shared markers for dysfunction of the heart and kidneys.

### Cystatin C

High levels of Cystatin C were strongly associated with an increased risk of renal dysfunction in all three EV sub‐fractions in our study. The same was true for plasma Cystatin C. This was expected because Cystatin C is known as a marker for renal failure with higher levels of Cystatin C corresponding with more renal dysfunction.^11^ Even more interestingly, we found that Cystatin C was associated with heart failure in all three sub‐fractions and in plasma as well. Although Cystatin C was more potent marker for renal dysfunction, our study showed that the association for EV and plasma levels of Cystatin C with heart failure remained significant in patients with normal GFR levels. This supports the hypothesis that Cystatin C may be a marker for heart failure independently of renal function, as described previously.^20^ The results of our study are in line with previously published elevated risk of (future) cardiovascular events and mortality in patients with high plasma values of Cystatin C^12^, ^21^, ^22^, ^23^ and high vesicle levels of Cystatin C.^8^ In the current study, we found no difference between EV and plasma levels of Cystatin C, suggesting that the protein itself and not specific EV Cystatin C is important in the combined organ failure of the CRS.

### CD14

CD14 is important in innate immune system as co‐receptor for different toll‐like receptors.^13^ It is a pro‐inflammatory protein, and high levels of CD14 both in plasma and in EVs have been associated with increased risk of cardiovascular events and mortality in previous studies.^8^, ^24^ Similarly, we found an increased risk of both heart failure and renal dysfunction in patients with higher EV CD14 levels. In this, our multinomial model showed the highest OR for patients with both heart failure and renal dysfunction, indicating that elevated EV CD14 levels associate with higher risk of the concurrence of both conditions than with either heart failure or renal dysfunction in isolation. This was not found for plasma CD14 levels, identifying a specific role for CD14 in EVs in the pathophysiology of both heart failure and renal dysfunction. CD14 is a membrane anchored protein with also a soluble form. This is in contrast to Cystatin C, which has no membrane‐binding domains. Although we cannot discriminate between soluble and membrane CD14 in our immunoassay, our data suggest that EV‐bound membrane CD14 and not the soluble plasma CD14 is important in heart failure and kidney dysfunction.

### SerpinG1 and SerpinF2

SerpinG1 is an acute‐phase protein that regulates complement activation and SerpinF2 reduces fibrinolysis by inhibition of plasmin.^14^, ^15^ In the current study, we found that both SerpinG1 and SerpinF2 were associated with heart failure. Our multinomial models showed an association for SerpinG1 in the LDL and TEX sub‐fractions and in plasma with heart failure and concurrent heart failure and renal dysfunction, but not with renal dysfunction alone. Similarly, SerpinF2 levels in LDL EV sub‐fraction and in plasma were associated with heart failure, but there was no association between SerpinF2 and renal dysfunction in any EV sub‐fraction or in plasma. According to these results, SerpinG1 and SerpinF2 seem to be markers for heart failure rather than for renal dysfunction, suggesting that the complement and fibrinolysis pathways may be more important pathophysiologically in development of heart failure than renal dysfunction. Interestingly, patients with heart failure have lower SerpinF2 levels as compared with those without heart failure. Because SerpinF2 reduces fibrinolysis by inhibiting plasmin, this suggests that there may be increased fibrinolytic activity in patients with heart failure; however, how/if this relates to the relative risks of bleeding versus thrombosis in heart failure remains to be investigated.

### Common denominators for renal and cardiac dysfunction

In the CRS, dysfunction of the heart or kidneys may induce dysfunction of the other organ.^2^ Conventionally, the main underlying mechanism of CRS has been thought to be impaired renal blood flow as result of cardiac dysfunction. However, CRS is bidirectional and involves complex interactions between the heart and kidneys.^1^, ^2^, ^25^ Not only hemodynamic but also neural, hormonal, biochemical, and immunologic factors play a role in the pathophysiology of CRS.^1^, ^2^, ^25^ Inflammation may be crucial in this process, as atherosclerosis is an inflammatory process and inflammation plays a role in aetiology of both heart failure and renal dysfunction.^25^, ^26^ In the current study, vesicle levels of pro‐inflammatory CD14 were elevated in patients with heart failure, renal dysfunction, and the combination of both conditions. This suggests that CD14 in EVs may reflect toll‐like receptor involvement in both heart failure and renal dysfunction, although the exact underlying mechanisms of this finding remain unclear. One possible explanation is that a general increased inflammatory status, including elevated EV CD14 levels, as result of cell injury in patients with classic risk factors, contributes to development of dysfunction of both the heart and kidneys. EVs enriched with CD14 are possibly not cleared as well in patients with impaired renal function as compared with those with normal GFR levels. Circulating CD14 EVs may then increase inflammatory processes in the blood vessels and heart, resulting in higher risk of heart failure. Our data can only be hypothesis generating and need to be further validated and mechanisms need to be explored in future studies.

In addition to CD14, we found that Cystatin C both in EVs and in plasma was associated with both renal dysfunction and heart failure. Cystatin C is a known marker of renal dysfunction^11^ but was also found to be an independent risk factor for heart failure in older adults.^27^ How Cystatin C associates with risk of heart failure in patients with normal renal function remains unclear, but other reports suggest that again inflammation may an important linking mechanism.^28^

Identifying common markers for heart failure and renal dysfunction, such as EV CD14 and Cystatin C, is of interest because of the multifactorial feedback pathways linking the heart and kidneys. It contributes to our understanding of the pathophysiology of CRS and provides possible targets for future investigation hopefully leading to effective intervention or prevention strategies.

### Strengths and limitations

Strengths of this study include the fact that our data were collected prospectively and that the total study population consisted of patients with dyspnoea. This means that our controls (patients with no renal dysfunction and no heart failure) were not healthy controls but similarly symptomatic patients compared with those with heart failure or renal dysfunction. Furthermore, we used plasma samples to analyse the EV protein levels, which is preferred above serum samples because the process of serum collection may cause vesicle release due to activation of platelets and other cells.

This study also has several limitations. There could be some misclassification of heart failure diagnosis as consequence of blinding of the two assessors to NT‐proBNP levels. However, analyses of the association between protein levels and NT‐proBNP levels showed similar results as the results of analyses where heart failure was used as outcome (*Table* *S1*), suggesting that the effect of possible misclassification is limited. Another limitation is the fact that we only have measurements of proteins at one point in time. To investigate causal relationships, it is preferred to perform a follow‐up study over longer period of time with multiple blood sample measurements in association with other mechanistic experiments. Finally, we corrected for most important risk factors for cardiovascular diseases, but an uncertain potential for residual confounding remains.

In short, more research is needed to examine the mechanisms underlying the observed results and to investigate the origin of the EVs. However, this study is the first indication that EV levels of CD14 and Cystatin C may be common markers for heart failure and renal dysfunction.

## Conclusions

EV levels of CD14 and Cystatin C are associated with both renal dysfunction and heart failure in patients presenting with dyspnoea at the emergency department. These data suggest that EV proteins may be involved in the combined organ failure of the CRS and may represent possible targets for prevention or treatment.

## Conflict of interest

None declared.

## Funding

This work was supported by Start‐up grant National University of Singapore to D.P.V.K; National Medical Research Council Center Grant (http://www.nmrc.gov.sg/content/nmrc_internet/home/grant‐navigation/competitive‐research‐grants/CG.html) to D.P.V.K., A.M.R., and C.S.P.L.; ATTRaCT SPF grant (https://www.a‐star.edu.sg/Scholarships/Overview.aspx) to D.P.V.K., A.M.R., and C.S.P.L.; National Medical Research Council CS‐IRG (http://www.nmrc.gov.sg/content/nmrc_internet/home/grant‐navigation/competitive‐research‐grants/cs‐individual‐research‐grant.html) to D.P.V.K. and C.S.P.L.; Queen of Hearts programme Dutch Heart Foundation (https://www.hartstichting.nl/home) to D.P.V.K.; and KNAW strategic grant (https://www.knaw.nl/ens) to D.P.V.K.

## Supporting information


**Table S1**. Association between EV and plasma protein levels and NT‐proBNP levels.
**Table S2**. Association between EV and plasma protein levels and GFR.
**Table S3**. Multinomial regression analyses.

## References

[1] Bock JS , Gottlieb SS . Cardiorenal syndrome: new perspectives. Circulation 2010; 121: 2592–2600. 20547939 10.1161/CIRCULATIONAHA.109.886473

[2] Ronco C , Haapio M , House AA , Anavekar N , Bellomo R . Cardiorenal syndrome. J Am Coll Cardiol 2008; 52: 1527–1539. 19007588 10.1016/j.jacc.2008.07.051

[3] Damman K , Navis G , Voors AA , Asselbergs FW , Smilde TDJ , Cleland JGF , van Veldhuisen DJ , Hillege HL . Worsening renal function and prognosis in heart failure: systematic review and meta‐analysis. J Card Fail 2007; 13: 599–608. 17923350 10.1016/j.cardfail.2007.04.008

[4] McAlister FA , Ezekowitz J , Tonelli M , Armstrong PW . Renal insufficiency and heart failure: prognostic and therapeutic implications from a prospective cohort study. Circulation 2004; 109: 1004–1009. 14769700 10.1161/01.CIR.0000116764.53225.A9

[5] Loyer X , Vion AC , Tedgui A , Boulanger CM . Microvesicles as cell–cell messengers in cardiovascular diseases. Circ Res 2014; 114: 345–353. 24436430 10.1161/CIRCRESAHA.113.300858

[6] Bank IE , Timmers L , Gijsberts CM , Zhang Y‐N , Mosterd A , Wang J‐W , Chan MY , de Hoog V , Lim SK , Sze SK , Lam CS , de Kleijn DP . The diagnostic and prognostic potential of plasma extracellular vesicles for cardiovascular disease. Expert Rev Mol Diagn 2015; 15: 1577–1588. 26535492 10.1586/14737159.2015.1109450

[7] Wang JW , Gijsberts CM , Seneviratna A , de Hoog VC , Vrijenhoek JE , Schoneveld AH , Chan MY , Lam CS , Richards AM , Lee CN , Mosterd A , Sze SK , Timmers L , Lim SK , Pasterkamp G , de Kleijn DP . Plasma extracellular vesicle protein content for diagnosis and prognosis of global cardiovascular disease. Neth Hear J 2013; 21: 467–471. 10.1007/s12471-013-0462-3 PMC3776081 23975618

[8] Kanhai DA , Visseren FL , van der Graaf Y , Schoneveld AH , Catanzariti LM , Timmers L , Kappelle LJ , Uiterwaal CS , Lim SK , Sze SK , Pasterkamp G , de Kleijn DP , Group SS . Microvesicle protein levels are associated with increased risk for future vascular events and mortality in patients with clinically manifest vascular disease. Int J Cardiol 2013; 168: 2358–2363. 23484740 10.1016/j.ijcard.2013.01.231

[9] Virzì GM , Clementi A , Ronco C . Cellular apoptosis in the cardiorenal axis. Heart Fail Rev 2016; 21: 177–189. 26852141 10.1007/s10741-016-9534-y

[10] Zhang YN , Vernooij F , Ibrahim I , Ooi S , Gijsberts CM , Schoneveld AH , Sen KW , den Ruijter HM , Timmers L , Richards AM , Jong CT , Mazlan I , Wang JW , Lam CSP , de Kleijn DPV . Extracellular vesicle proteins associated with systemic vascular events correlate with heart failure: an observational study in a dyspnoea cohort. PLoS ONE 2016; 11: 1–19. 10.1371/journal.pone.0148073 PMC4731211 26820481

[11] Dharnidharka VR , Kwon C , Stevens G . Serum cystatin C is superior to serum creatinine as a marker of kidney function: a meta‐analysis. Am J Kidney Dis National Kidney Foundation, Inc 2002; 40: 221–226. 12148093 10.1053/ajkd.2002.34487

[12] Shlipak MG , Sarnak MJ , Katz R , Fried LF , Seliger SL , Newman AB , Siscovick DS , Stehman‐Breen C . Cystatin C and the risk of death and cardiovascular events among elderly persons. N Engl J Med 2005; 352: 2049–2060. 15901858 10.1056/NEJMoa043161

[13] Zanoni I , Granucci F . Role of CD14 in host protection against infections and in metabolism regulation. Front Cell Infect Microbiol 2013; 3: 1–6. 23898465 10.3389/fcimb.2013.00032 PMC3721004

[14] Cicardi M , Zingale L , Zanichelli A , Pappalardo E , Cicardi B . C1 inhibitor: molecular and clinical aspects. sSpringer Semin Immunopathol 2005; 27: 286–298. 10.1007/s00281-005-0001-4 16267649

[15] Abdul S , Leebeek FWG , Rijken DC , Uitte De Willige S . Natural heterogeneity of a2‐antiplasmin: functional and clinical consequences. Blood 2016; 127: 538–545. 26626994 10.1182/blood-2015-09-670117

[16] Wang JW , Zhang YN , Sze SK , van de Weg SM , Vernooij F , Schoneveld AH , Tan SH , Versteeg HH , Timmers L , Lam CS , de Kleijn DP . Lowering low‐density lipoprotein particles in plasma using dextran sulphate co‐precipitates procoagulant extracellular vesicles. Int J Mol Sci 2017; 19: E94. 29286309 10.3390/ijms19010094 PMC5796044

[17] Mcmurray JJV , Adamopoulos S , Anker SD , Auricchio A , Böhm M , Dickstein K , Falk V , Filippatos G , Fonseca C , Gomez‐Sanchez MA , Jaarsma T , Kober L , Lip GYH , Pietro MA , Parkhomenko A , Pieske BM , Popescu BA , Ronnevik PK , Rutten FH , Schwitter J , Seferovic P , Stepinska J , Trindade PT , Voors AA , Zannad F , Zeiher A , Bax JJ , Baumgartner H , Ceconi C , Dean V . ESC Guidelines for the diagnosis and treatment of acute and chronic heart failure 2012. Eur J Heart Fail 2012; 14: 803–869. 22828712 10.1093/eurjhf/hfs105

[18] Ibrahim I , Sen KW , Frampton C , Troughton R , Liew OW , Chong JPC , Chan SP , Tan LL , Lin WQ , Pemberton CJ , Ooi SBS , Richards AM . Superior performance of N‐terminal pro brain natriuretic peptide for diagnosis of acute decompensated heart failure in an Asian compared with a Western setting. Eur J Heart Fail 2016; 19: 209–217. 27620387 10.1002/ejhf.612

[19] R‐Foundation . R: A language and environment for statistical computing. Available from https://www.r‐project.org/. 2018.

[20] Levin A , Lan JH . Cystatin C and cardiovascular disease: causality, association, and clinical implications of knowing the difference. J Am Coll Cardiol Elsevier 2016; 68: 946–948. 27561769 10.1016/j.jacc.2016.06.037

[21] Lee M , Saver JL , Huang WH , Chow J , Chang KH , Ovbiagele B . Impact of elevated cystatin C level on cardiovascular disease risk in predominantly high cardiovascular risk populations: a meta‐analysis. Circ Cardiovasc Qual Outcomes 2010; 3: 675–683. 20923994 10.1161/CIRCOUTCOMES.110.957696

[22] Arimoto T , Takeishi Y , Niizeki T , Takabatake N , Okuyama H , Fukui A , Tachibana H , Nozaki N , Hirono O , Tsunoda Y , Miyashita T , Shishido T , Takahashi H , Koyama Y , Kubota I . Cystatin C, a novel measure of renal function, is an independent predictor of cardiac events in patients with heart failure. J Card Fail 2005; 11: 595–601. 16230262 10.1016/j.cardfail.2005.06.001

[23] Ix JH , Shlipak MG , Chertow GM , Whooley MA . Association of cystatin C with mortality, cardiovascular events, and incident heart failure among persons with coronary heart disease: data from the Heart and Soul Study. Circulation 2007; 115: 173–179. 17190862 10.1161/CIRCULATIONAHA.106.644286 PMC2771187

[24] Reiner AP , Lange EM , Jenny NS , Chaves PHM , Ellis J , Li J , Walston J , Lange LA , Cushman M , Tracy RP . Soluble CD14: genomewide association analysis and relationship to cardiovascular risk and mortality in older adults. Arterioscler Thromb Vasc Biol 2013; 33: 158–164. 23162014 10.1161/ATVBAHA.112.300421 PMC3826541

[25] Kingma JS , Simard D , Rouleau JR , Drolet B , Simard C . The physiopathology of cardiorenal syndrome: a review of the potential contributions of inflammation. J Cardiov Dev Dis 2017; 4: pii E21. 10.3390/jcdd4040021 PMC5753122 29367550

[26] Tuttolomondo A , di Raimondo D , Pecoraro R , Arnao V , Pinto A , Licata G . Atherosclerosis: an inflammatory disease. Curr Pharm Des 2012; 18: 4266–4288. 22390643 10.2174/138161212802481237

[27] Sarnak MJ , Katz R , Stehman‐Breen CO , Fried LF , Jenny NS , Psaty BM , Newman AB , Siscovick D , Shlipak MG , the Cardiovascular Health Study . Cystatin C concentration as a risk factor for heart failure in older adults. Ann Intern Med 2005; 142: 497–505. 15809461 10.7326/0003-4819-142-7-200504050-00008

[28] Okura T , Jotoku M , Irita J , Enomoto D , Nagao T , Desilva VR , Yamane S , Pei Z , Kojima S , Hamano Y , Mashiba S , Kurata M , Miyoshi KI , Higaki J . Association between cystatin C and inflammation in patients with essential hypertension. Clin Exp Nephrol 2010; 14: 584–588. 20809110 10.1007/s10157-010-0334-8

