# Supplementary material for: Extracellular vesicle Cystatin C and CD14 are associated with both renal dysfunction and heart failure
Source: ESC Heart Fail. 2020 Jul 10;7(5):2240–9. doi: 10.1002/ehf2.12699 (PMC7524227; doi:10.1002/ehf2.12699)
Supplement: Supplementary file 1 — Table S1. Association between EV and plasma protein levels and NT‐proBNP levels. Table S2. Association between EV and plasma protein levels and GFR. Table S3. Multinomial regression analyses. [file EHF2-7-2240-s001.docx]

**SUPPLEMENTAL MATERIAL**

**Original manuscript: Extracellular vesicle CystatinC and CD14 are associated with both renal dysfunction and heart failure**

Corresponding author: Dominique P.V. de Kleijn, email: [d.p.v.dekleijn@umcutrecht.nl](mailto:dkleijn@umcutrecht.nl)

**Content**

[Supplemental tables 2](#_Toc536792112)

[Table S1. Association between EV and plasma protein levels and NT-proBNP levels 2](#_Toc536792113)

[Table S2. Association between EV and plasma protein levels and GFR 3](#_Toc536792114)

[Table S3. Multinomial regression analyses 4](#_Toc536792115)

# Supplemental tables

## Table S1. Association between EV and plasma protein levels and NT-proBNP levels

|  |  | **Univariable** | | | |  | **Multivariable*** | | | |
| --- | --- | --- | --- | --- | --- | --- | --- | --- | --- | --- |
|  |  | **Beta** | **97.5% CI** | | **p-value** |  | **Beta** | **97.5% CI** | | **p-value** |
| **CystatinC** |  |  |  |  |  |  |  |  |  |  |
| HDL |  | **1.19** | **0.95** | **1.42** | **<0.001** |  | **0.72** | **0.48** | **0.96** | **<0.001** |
| LDL |  | **0.88** | **0.63** | **1.13** | **<0.001** |  | **0.53** | **0.30** | **0.77** | **<0.001** |
| TEX |  | **1.19** | **0.95** | **1.42** | **<0.001** |  | **0.74** | **0.50** | **0.99** | **<0.001** |
| Plasma |  | **0.81** | **0.56** | **1.06** | **<0.001** |  | **0.471** | **0.24** | **0.70** | **<0.001** |
| **CD14** |  |  |  |  |  |  |  |  |  |  |
| HDL |  | **0.90** | **0.65** | **1.15** | **<0.001** |  | **0.55** | **0.33** | **0.78** | **<0.001** |
| LDL |  | **0.44** | **0.17** | **0.70** | **<0.001** |  | **0.32** | **0.09** | **0.55** | **0.002** |
| TEX |  | **0.71** | **0.46** | **0.97** | **<0.001** |  | **0.49** | **0.26** | **0.72** | **<0.001** |
| Plasma |  | -0.10 | -0.38 | 0.18 | 0.419 |  | -0.09 | -0.33 | 0.15 | 0.407 |
| **SerpinG1** |  |  |  |  |  |  |  |  |  |  |
| HDL |  | -0.12 | -0.39 | 0.15 | 0.332 |  | -0.11 | -0.33 | 0.12 | 0.291 |
| LDL |  | **-0.75** | **-1.01** | **-0.50** | **<0.001** |  | **-0.44** | **-0.67** | **-0.21** | **<0.001** |
| TEX |  | **0.33** | **0.07** | **0.60** | **0.005** |  | **0.31** | **0.09** | **0.53** | **0.002** |
| Plasma |  | **0.47** | **0.19** | **0.74** | **<0.001** |  | **0.40** | **0.17** | **0.64** | **<0.001** |
| **SerpinF2** |  |  |  |  |  |  |  |  |  |  |
| HDL |  | 0.04 | -0.23 | 0.31 | 0.736 |  | -0.06 | -0.29 | 0.17 | 0.572 |
| LDL |  | **-0.27** | **-0.54** | **0.00** | **0.023** |  | -0.21 | -0.44 | 0.02 | 0.038 |
| TEX |  | -0.01 | -0.28 | 0.26 | 0.959 |  | -0.06 | -0.29 | 0.17 | 0.568 |
| Plasma |  | **-0.33** | **-0.61** | **-0.05** | **0.008** |  | **-0.24** | **-0.48** | **0.00** | **0.023** |

Results of univariable and multivariable linear regression analyses for the association between CystatinC, CD14, SerpinG1 and SerpinF2 levels (in HDL, LDL and TEX sub-fractions and in plasma) and NT-proBNP levels. All protein levels were standardised prior to analyses and NT-proBNP levels underwent logarithmic transformation. Shown betas therefore represent increase in log(NT-proBNP) for one standard deviation increase in the corresponding protein level. Values in bold are statistically significant (p<0.025).

* corrected for age, gender, ethnicity, diabetes, hypertension and ischemic heart disease.

Abbreviations: NT-proBNP: N-terminal pro brain natriuretic peptide; EV: extracellular vesicle; Beta: Beta coefficient; 97.5% CI: 97.5% confidence interval. HDL: High-density lipoprotein; LDL: Low-density lipoprotein; TEX: Total extracellular vesicles.

## Table S2. Association between EV and plasma protein levels and GFR

|  |  | **Univariable** | | | |  | **Multivariable*** | | | |
| --- | --- | --- | --- | --- | --- | --- | --- | --- | --- | --- |
|  |  | **Beta** | **97.5% CI** | | **p-value** |  | **Beta** | **97.5% CI** | | **p-value** |
| **CystatinC** |  |  |  |  |  |  |  |  |  |  |
| HDL |  | **-19.59** | **-22.06** | **-17.12** | **<0.001** |  | **-13.98** | **-16.44** | **-11.51** | **<0.001** |
| LDL |  | **-13.21** | **-16.20** | **-10.22** | **<0.001** |  | **-8.91** | **-11.50** | **-6.32** | **<0.001** |
| TEX |  | **-21.08** | **-23.39** | **-18.77** | **<0.001** |  | **-15.87** | **-18.24** | **-13.50** | **<0.001** |
| Plasma |  | **-12.42** | **-15.42** | **-9.41** | **<0.001** |  | **-8.07** | **-10.61** | **-5.54** | **<0.001** |
| **CD14** |  |  |  |  |  |  |  |  |  |  |
| HDL |  | **-10.92** | **-13.99** | **-7.84** | **<0.001** |  | **-5.58** | **-8.25** | **-2.92** | **<0.001** |
| LDL |  | **-7.24** | **-10.47** | **-4.01** | **<0.001** |  | **-5.14** | **-7.78** | **-2.50** | **<0.001** |
| TEX |  | **-10.62** | **-13.73** | **-7.52** | **<0.001** |  | **-6.40** | **-9.06** | **-3.74** | **<0.001** |
| Plasma |  | 1.05 | -2.44 | 4.53 | 0.500 |  | 1.01 | -1.79 | 3.80 | 0.418 |
| **SerpinG1** |  |  |  |  |  |  |  |  |  |  |
| HDL |  | -1.71 | -5.05 | 1.62 | 0.248 |  | -1.19 | -3.84 | 1.45 | 0.311 |
| LDL |  | **8.28** | **5.07** | **11.49** | **<0.001** |  | **4.54** | **1.87** | **7.21** | **<0.001** |
| TEX |  | **-3.51** | **-6.82** | **-0.19** | **0.018** |  | -2.27 | -4.89 | 0.35 | 0.052 |
| Plasma |  | -2.11 | -5.57 | 1.35 | 0.171 |  | -0.99 | -3.78 | 1.80 | 0.426 |
| **SerpinF2** |  |  |  |  |  |  |  |  |  |  |
| HDL |  | 0.64 | -2.69 | 3.96 | 0.666 |  | 1.19 | -1.47 | 3.86 | 0.315 |
| LDL |  | 2.53 | -0.79 | 5.85 | 0.087 |  | 1.26 | -1.44 | 3.95 | 0.294 |
| TEX |  | -0.36 | -3.71 | 2.98 | 0.808 |  | 0.04 | -2.66 | 2.73 | 0.975 |
| Plasma |  | 3.00 | -0.46 | 6.47 | 0.052 |  | 1.68 | -1.08 | 4.43 | 0.172 |

Results of univariable and multivariable linear regression analyses for the association between CystatinC, CD14, SerpinG1 and SerpinF2 levels (in HDL, LDL and TEX sub-fractions and in plasma) and GFR. All protein levels were standardised prior to analyses, shown betas therefore represent increase in GFR for one standard deviation increase in the corresponding protein level. Values in bold are statistically significant (p<0.025).

* corrected for age, gender, ethnicity, diabetes, hypertension and ischemic heart disease.

Abbreviations: GFR: Glomerular filtration rate; EV: extracellular vesicle; Beta: Beta coefficient; 97.5%CI: 97.5% confidence interval. HDL: High-density lipoprotein; LDL: Low-density lipoprotein; TEX: Total extracellular vesicles.

## Table S3. Multinomial regression analyses

|  | **Univariable** | | |  | **Multivariable*** | | |
| --- | --- | --- | --- | --- | --- | --- | --- |
|  | **OR** | **95%CI** | |  | **OR** | **95%CI** | |
| **CystatinC HDL** |  |  |  |  |  |  |  |
| None | Ref | | |  | Ref | | |
| HF | **2.62** | **1.84** | **3.73** |  | **2.19** | **1.50** | **3.19** |
| RD | **9.32** | **5.37** | **16.16** |  | **7.68** | **4.18** | **14.12** |
| HF+RD | **10.87** | **6.53** | **18.10** |  | **8.38** | **4.69** | **14.95** |
| **CystatinC LDL** |  |  |  |  |  |  |  |
| None | Ref | | |  | Ref | | |
| HF | **1.86** | **1.38** | **2.49** |  | **1.70** | **1.23** | **2.36** |
| RD | **3.63** | **2.28** | **5.78** |  | **4.04** | **2.38** | **6.87** |
| HF+RD | **4.20** | **2.81** | **6.27** |  | **4.16** | **2.52** | **6.84** |
| **CystatinC TEX** |  |  |  |  |  |  |  |
| None | Ref | | |  | Ref | | |
| HF | **1.89** | **1.34** | **2.68** |  | **1.56** | **1.07** | **2.29** |
| RD | **10.42** | **5.85** | **18.55** |  | **6.73** | **3.66** | **12.36** |
| HF+RD | **12.90** | **7.47** | **22.27** |  | **8.50** | **4.66** | **15.49** |
| **CystatinC Plasma** |  |  |  |  |  |  |  |
| None | Ref | | |  | Ref | | |
| HF | **1.93** | **1.31** | **2.85** |  | **1.54** | **1.09** | **2.18** |
| RD | **4.88** | **2.98** | **7.99** |  | **3.52** | **2.11** | **5.86** |
| HF+RD | **5.40** | **3.46** | **8.45** |  | **3.37** | **2.09** | **5.45** |
| **CD14 HDL** |  |  |  |  |  |  |  |
| None | Ref | | |  | Ref | | |
| HF | **1.69** | **1.27** | **2.24** |  | **1.57** | **1.16** | **2.13** |
| RD | **2.14** | **1.42** | **3.24** |  | **1.87** | **1.18** | **2.96** |
| HF+RD | **3.33** | **2.31** | **4.82** |  | **2.78** | **1.78** | **4.32** |
| **CD14 LDL** |  |  |  |  |  |  |  |
| None | Ref | | |  | Ref | | |
| HF | 1.20 | 0.92 | 1.56 |  | 1.22 | 0.91 | 1.63 |
| RD | **1.87** | **1.28** | **2.72** |  | **1.78** | **1.18** | **2.70** |
| HF+RD | **1.70** | **1.25** | **2.31** |  | **1.60** | **1.09** | **2.37** |

## Table S3 continued

|  | **Univariable** | | |  | **Multivariable*** | | |
| --- | --- | --- | --- | --- | --- | --- | --- |
|  | **OR** | **95%CI** | |  | **OR** | **95%CI** | |
| **CD14 TEX** |  |  |  |  |  |  |  |
| None | Ref | | |  | Ref | | |
| HF | 1.30 | 0.99 | 1.71 |  | 1.32 | 0.97 | 1.78 |
| RD | **2.08** | **1.43** | **3.02** |  | **1.72** | **1.12** | **2.64** |
| HF+RD | **2.50** | **1.82** | **3.42** |  | **2.26** | **1.50** | **3.42** |
| **CD14 Plasma** |  |  |  |  |  |  |  |
| None | Ref | | |  | Ref | | |
| HF | 0.85 | 0.65 | 1.11 |  | **0.73** | **0.54** | **1.00** |
| RD | 0.85 | 0.59 | 1.24 |  | 0.86 | 0.59 | 1.27 |
| HF+RD | 0.81 | 0.60 | 1.09 |  | 0.81 | 0.55 | 1.18 |
| **SerpinG1 HDL** |  |  |  |  |  |  |  |
| None | Ref | | |  | Ref | | |
| HF | 0.80 | 0.63 | 1.03 |  | 0.81 | 0.61 | 1.08 |
| RD | 0.98 | 0.68 | 1.41 |  | 0.95 | 0.63 | 1.42 |
| HF+RD | 1.03 | 0.77 | 1.38 |  | 0.98 | 0.69 | 1.41 |
| **SerpinG1 LDL** |  |  |  |  |  |  |  |
| None | Ref | | |  | Ref | | |
| HF | **0.66** | **0.50** | **0.88** |  | **0.68** | **0.50** | **0.92** |
| RD | **0.62** | **0.42** | **0.93** |  | 0.68 | 0.44 | 1.05 |
| HF+RD | **0.40** | **0.28** | **0.56** |  | **0.48** | **0.32** | **0.72** |
| **SerpinG1 TEX** |  |  |  |  |  |  |  |
| None | Ref | | |  | Ref | | |
| HF | 1.28 | 0.99 | 1.65 |  | **1.41** | **1.07** | **1.85** |
| RD | 1.08 | 0.74 | 1.56 |  | 0.98 | 0.63 | 1.50 |
| HF+RD | **1.53** | **1.15** | **2.05** |  | **1.66** | **1.14** | **2.42** |
| **SerpinG1 Plasma** |  |  |  |  |  |  |  |
| None | Ref | | |  | Ref | | |
| HF | **2.30** | **1.69** | **3.12** |  | **2.35** | **1.68** | **3.28** |
| RD | 1.20 | 0.78 | 1.83 |  | 1.31 | 0.84 | 2.05 |
| HF+RD | **1.80** | **1.28** | **2.52** |  | **1.88** | **1.26** | **2.80** |

## Table S3 continued

|  | **Univariable** | | |  | **Multivariable*** | | |
| --- | --- | --- | --- | --- | --- | --- | --- |
|  | **OR** | **95%CI** | |  | **OR** | **95%CI** | |
| **SerpinF2 HDL** |  |  |  |  |  |  |  |
| None | Ref | | |  | Ref | | |
| HF | 1.01 | 0.79 | 1.30 |  | 1.00 | 0.76 | 1.30 |
| RD | 1.11 | 0.77 | 1.60 |  | 1.11 | 0.74 | 1.68 |
| HF+RD | 0.94 | 0.70 | 1.25 |  | 0.95 | 0.67 | 1.36 |
| **SerpinF2 LDL** |  |  |  |  |  |  |  |
| None | Ref | | |  | Ref | | |
| HF | **0.71** | **0.55** | **0.91** |  | **0.73** | **0.56** | **0.96** |
| RD | 1.03 | 0.70 | 1.51 |  | 1.05 | 0.67 | 1.64 |
| HF+RD | **0.71** | **0.54** | **0.95** |  | 0.77 | 0.53 | 1.13 |
| **SerpinF2 TEX** |  |  |  |  |  |  |  |
| None | Ref | | |  | Ref | | |
| HF | **0.78** | **0.61** | **1.00** |  | 0.79 | 0.60 | 1.04 |
| RD | 1.15 | 0.78 | 1.68 |  | 1.07 | 0.70 | 1.64 |
| HF+RD | 1.00 | 0.74 | 1.34 |  | 1.04 | 0.71 | 1.51 |
| **SerpinF2 Plasma** |  |  |  |  |  |  |  |
| None | Ref | | |  | Ref | | |
| HF | **0.67** | **0.50** | **0.89** |  | **0.63** | **0.47** | **0.85** |
| RD | 0.78 | 0.53 | 1.15 |  | 0.81 | 0.52 | 1.26 |
| HF+RD | **0.71** | **0.52** | **0.97** |  | **0.64** | **0.42** | **0.97** |

Results of univariable and multivariable multinomial regression analyses for the association between protein levels and heart failure, renal dysfunction and the combination of both conditions. Odds ratios with corresponding 95% confidence intervals are shown for CystatinC, CD14, SerpinG1 and SerpinF2 levels in HDL, LDL and TEX sub-fractions and in plasma. All protein levels were standardised prior to analyses, shown odds ratios therefore represent odds ratios for one standard deviation increase in the corresponding protein level. Patients who did not have heart failure nor renal dysfunction were chosen as reference category. Values in bold are statistically significant (p<0.05).

* corrected for age, gender, ethnicity, diabetes, hypertension and ischemic heart disease.

Abbreviations: HF: heart failure; RD: renal dysfunction; OR: odds ratio; 95%CI: 95% confidence interval; HDL: High-density lipoprotein; LDL: Low-density lipoprotein; TEX: Total extracellular vesicles.
